# Temporal trends in respiratory and gastrointestinal infections at the Grand Magal of Touba: the effect of the COVID-19 pandemic

**DOI:** 10.1016/j.nmni.2025.101688

**Published:** 2025-12-15

**Authors:** Coumba Diouf, Ihssane Ouaddane, Ndiaw Goumballa, Déguéne Fam, Hubert Bassene, Cheikh Sokhna, Philippe Gautret

**Affiliations:** a Aix Marseille Univ, IRD, AP-HM, SSA, RITMES, Marseille, France; b Aix Marseille Univ, IRD, AP-HM, SSA, MINES, Marseille, France; c IHU-Méditerranée Infection, Marseille, France; d IRD, UCAD, MINES, Dakar, Senegal

**Keywords:** Mass gathering, Temporal trends, Respiratory, Gastrointestinal, COVID-19, Grand Magal of Touba, Senegal

## Abstract

**Background:**

The COVID-19 pandemic has been associated with changes in the epidemiology of other respiratory tract and gastrointestinal Infections.

**Methods:**

Respiratory and gastrointestinal pathogen carriage was assessed by qPCR between 2017 and 2024 in 1327 participants comprising 837 pilgrims from villages in South Senegal and 490 patients consulting for respiratory symptoms at the Mbacké health care centre near Touba.

**Results:**

Among pilgrims returning from the GMT, the overall prevalence of respiratory viruses significantly decreased during the pandemic years in Senegal (2020–2021). This was mostly due to rhinovirus and the prevalence of variations of endemic coronaviruses. In contrast, the prevalence of influenza viruses increased during the pandemic years. Overall, the prevalence of respiratory bacteria decreased during and after the pandemic, with the notable exception of *Haemophilus* spp. The prevalence of gastrointestinal bacterial pathogens decreased during the pandemic, which was mostly due to a decrease in various *Escherichia coli* pathotypes,.

**In mbacké patients:**

influenza A virus was the most common virus, and its prevalence was significantly higher during and after the pandemic, while the prevalence of other respiratory viruses decreased over time from 2018 until 2024. Infections with *Streptococcus pneumoniae* and *Moraxella catarrhalis* increased during the COVID-19 period, while that of *S. aureus* decreased.

**Conclusion:**

Significant variations in the prevalence of respiratory and gastrointestinal pathogens were observed among pilgrims participating in the GMT during the COVID-19 pandemic in Senegal. This could be due to changes in individual non-pharmaceutical preventive measures during the pandemic period as well as changes in the demographic profile of participants.

## 1 Introduction

The Grand Magal de Touba (GMT) is considered the largest mass gathering in West Africa with between four and five million pilgrims taking part originating from Senegal and beyond. It is a religious event commemorating the departure into exile of Sheikh Ahmadou Bamba Mbacké, the founder of Mouridism, to Gabon in 1895. It is celebrated every year on Safar 18 in Touba, in the Mbacké department of Senegal. The crowded conditions that arise during this event are likely to increase the risk of transmission of infectious diseases, including febrile illnesses, diarrhoeal diseases, and respiratory infections, [1]. One study carried out during the GMT in 2016 showed that among 20 850 patients, headaches were the most frequent symptoms (28.2 %), followed by gastrointestinal symptoms (22.0 %), fever (17.2 %), and respiratory symptoms (17.1 %) [2].

The results of a study we carried out on pilgrim cohorts between 2017 and 2021 including 535 participants, showed that 55 % had respiratory symptoms and had acquired *Haemophilus spp*. (18.9 %), *Streptococcus pneumoniae* (14.1 %), *Klebsiella pneumoniae* (13 %), *Staphylococcus aureus* (12 %), rhinovirus (10 %), endemic coronaviruses (6 %), and *Moraxella catarrhalis* (5 %). A further 13 % of pilgrims reported gastrointestinal symptoms linked to the acquisition of *Enteroaggregative Escherichia coli* (EAEC) (18.9 %), *Enteropathogenic E. coli* (EPEC) (10.5 %), and *Enterohemorrhagic, E*. *coli* (EHEC) (7.4 %) [3]. Another study was conducted on ill pilgrims consulting at the Mbacké health care centre not far from Touba, where a high prevalence of respiratory pathogens was noted among patients suffering from respiratory symptoms, including *Haemophilus* spp. (73 %), *S. pneumoniae* (51 %), *M. catarrhalis* (46 %), *S. aureus* (22 %), influenza A virus (26 %), respiratory syncytial virus (16 %), rhinovirus (8 %), and influenza B virus (7 %) [4]. In December 2019, a new coronavirus (SARS-CoV-2) causing a series of cases of pneumonia was reported in the city of Wuhan, China [5]. Senegal had its first suspected case of COVID-19 on February 26, 2020 and confirmed an imported case on 2 March, followed by two more imported cases on 3 March and a fourth on 4 March [6]. The temporal evolution of the COVID-19 pandemic in Senegal (from March 2, 2020, to September 31, 2021) was marked by three waves in which 73 782 RT-PCR-confirmed cases and 1859 deaths were recorded. The first wave ran from March to November 2020. During this episode, the maximum number of confirmed cases (3371) was observed in August, and the maximum number of deaths (93) in July. Two peaks were observed during the second wave, which began in December 2020 and ended in May 2021. A first peak occurred in January with 7563 confirmed cases, then a second peak in February with 7805 confirmed cases and a maximum number of deaths (242) in February. The final wave ran from June to September 2021, with a peak in July (19 739 confirmed cases) and a maximum of deaths (407) recorded in August [7].

The aim of this study is to describe in temporal terms the results we obtained concerning GMT participants suffering from respiratory symptoms or gastrointestinal symptoms between 2017 and 2024 and to assess the potential role of the COVID-19 pandemic.

## 2 Materials and methods

### 2.1 Study population

The study involved cohorts of pilgrims departing from the villages of Dielmo (13°43′ 22.07″ N, 16°24′40.09″ W) and Ndiop (13°41′08.01″N, 16°23′01. 01″ W) located in southern Senegal, in the Fatick region (2017–2024) and pilgrims consulting for respiratory tract infection symptoms, at the Mbacké health care centre (14°47′56″N, 15°54′36″W) located 10 km from Touba (2018–2024). The Mbacké health care centre was chosen because of the high number of patients attending it during the five days of free GMT medical coverage, (1115 patients during the 2015 GMT, unpublished data). Pilgrims in the cohort were identified by the nurses in charge of the primary care centres in the two villages and participated in the study on a voluntary basis. Participants (or their parents if they were minors) were asked to sign a written consent form.

### 2.2 Data collection

For pilgrims in the cohort, the procedure was to collect data at the time of inclusion using a standardised questionnaire between seven and ten days before the patient's departure to Touba and six to nine days after their return. The survey included demographic data (age and sex), chronic diseases, use of individual preventive measures during the event, respiratory and gastrointestinal symptoms on returning from the pilgrimage, and hospitalisation rate. Respiratory and intestinal sampling was systematically proposed after participation in the GMT.

At the Mbacké health centre, during the five days of free GMT medical treatment, the medical team completed a demographic and clinical questionnaire, and patients were offered respiratory sampling.

### 2.3 Sample collection

Samples were taken using commercial rigid cotton swab applicators (Medical Wire & Equipment, Wiltshire, UK) inserted into the anterior nares or oropharynx and rectum, then placed in viral transport medium (Sigma Virocult®). Samples were kept at +4 °C before being transported to the Dakar laboratory for storage in a −80 °C freezer, then transferred to Marseille on dry ice for processing.

### 2.4 Identification of respiratory and gastrointestinal pathogens by PCR

To identify respiratory and gastrointestinal pathogens, we performed nucleic acid extraction (DNA and RNA) using the EZ1 Advanced XL (Qiagen, Hilden, Germany) with the Mini Virus v2.0 Kit (Qiagen) in accordance with the manufacturer's recommendations. Genes were amplified using the Light Cycler 480 Probes Master Kit (Roche Diagnostics, France) for bacteria and the Multiplex RNA Virus Master Kit (Roche Diagnostics, France) for viruses. A C1000 Touch thermal cycle (Bio-Rad, Hercules, CA, USA) was used to perform each real-time quantitative PCR. Negative controls (RNase-free water) and positive controls (DNA from a bacterial or viral strain) were included in all runs. Positive pathogen amplification results were evaluated against a cycle threshold (CT) value of ≤35. A panel of respiratory pathogens consisting of influenza A, influenza B, human rhinovirus, respiratory syncytial virus, SARS-CoV-2, human metapneumovirus, non-MERS human coronaviruses, human parainfluenzae viruses, adenovirus, *S. pneumoniae*, *S. aureus*, *Haemophilus* spp., *K. pneumoniae*, *Bordetella pertussis*, *Mycoplasma pneumoniae*, and *M. catarrhalis*. Gastrointestinal pathogens tested included astrovirus, norovirus, rotavirus, adenovirus, hepatitis A virus, hepatitis E virus, *G. lamblia*, *Entamoeba hystolitica*, *Cryptosporidium* spp, EPEC, EIEC, EAEC, EHEC, *Tropheryma whipplei*, and *Campylobacter jejuni*.

### Ethical approval

The protocol was approved by the National Ethics Committee for Health Research in Senegal (SEN 17/62). It was carried out in accordance with the good clinical practices recommended by the Declaration of Helsinki and its amendments.

## 3 Results

### 3.1 Characteristics of the study population (Table 1)

The study was conducted on a population of 1327 participants in GMTs held in November 2017, October 2018, 2019, and 2020, September 2021, 2022, and 2023, and August 2024.

Eight hundred and thirty-seven pilgrims were included in cohort studies including 304/837 from 2017 to 2019 (before the COVID-19 pandemic in Senegal), 231/837 pilgrims from 2020 to 2021 (during the pandemic), and 302/837 from 2022 to 2024 (after the pandemic). The study population was significantly younger during and after the pandemic, with a mean age of 23.4 and 24.4 years, than before (26.7 years) the pandemic. The proportion of female pilgrims was significantly higher after the pandemic (60.9 %) than before and after (50.0 %–48.9 %) and the proportion of pilgrims with chronic diseases decreased significantly during and after the pandemic (4.8 %–6.3 % vs. 11.8 %). A small proportion of pilgrims were vaccinated against COVID-19, at 5.6 % during the pandemic and 7.0 % after the pandemic. Similarly, a small proportion of pilgrims were vaccinated against invasive pneumococcal disease (PPV23) during the pandemic (5.2 %) and after the pandemic (13.3 %), with a significant increase over time. Vaccination rates against influenza were not systematically recorded, but between 2017 and 2021, only 0.2 % of pilgrims were vaccinated [3]. The frequent use of face masks significantly increased during the pandemic (48.5 %) compared to before the pandemic (4.3 %) and decreased to 2.3 % after the pandemic. Frequent hand washing increased during the pandemic (58.4 %), compared to before and after the pandemic (51.6 %–52.3 %) but these differences were not statistically significant. In contrast, the use of hand soap significantly increased during the pandemic (97.0 %) compared to before the pandemic (74.0 %) but decreased to a significantly lower rate after the pandemic (60.9 %). The length of stay at the GMT for pilgrims was significantly longer during and after the pandemic (4.4 days) than before (3.8 days).

At the Mbacké health centre, 490 patients with respiratory symptoms were included in the study, distributed as follows: 164/490 from 2018 to 2019 (before the COVID-19 pandemic), 162/490 from 2020 to 2021 (during the pandemic), and 164/490 from 2022 to 2024 (after the pandemic). The study population was significantly older after the pandemic, with a mean age of 22 years, than before and during the pandemic (18. and 13 years, respectively). The proportion of female patients was 58.6 %, with no significant variation over time, and the proportion of patients who had been vaccinated against COVID-19 was 1.2 % during the pandemic and increased significantly after the pandemic (12.8 %) (Table 2). Status regarding other preventive measures was not documented.

**Table 1:** Demographics, chronic conditions, and preventive measure in 837 pilgrims from Ndiop and Dielmo (2017–2024).

	Pre-pandemic period (2017–2019)	Peri-pandemic period (2020–2021)	Post-pandemic period (2022–2024)	Pre- vs. peri- (*P* value)	Pre-vs. post- (*P* value)	Peri- vs. post- (*P* value)
Age in years (mean, SD)	26.7	23.4	24.4	*P* = .0247	*P* = .0430	*P* = .8788
Sex (male %)	50.00	51.08	39.07	*P* = .436	*P* = .004	*P* = .004
Chronic diseases (%)	11.84	4.8	6.25	*P* = .003	*P* = .065	*P* = .365
Vaccination against COVID-19 (%)	0.00	5.63	6.95	*P* = .000	*P* = .332	*P* = .000
Vaccination against invasive pneumococcal disease: PPV23 (%)	0.00	5.19	13.3	*P* = .000	*P* = .000	*P* = .001
Face mask used more frequently than usual (%)	4.28	48.48	2.32	*P* = .000	*P* = .131	*P* = .000
Hand washing more frequently than usual (%)	51.64	58.44	52.32	*P* = .070	*P* = .466	*P* = .093
Use of hand soap	74.01	96.97	60.93	*P* = .000	*P* = .000	*P* = .000
Duration of stay in days (mean, SD)	3.8	4.4	4.4	*P* = .000	*P* = .000	*P* = .1510

**Table 2:** Demographics and COVID-19 vaccination status in 490 patients consulting at the Mbacké health care centre (2018–2024).

	Pre-pandemic period (2018–2019)	Peri-pandemic period (2020–2021)	Post-pandemic period (2022–2024)	Pre- vs. peri- (*P* value)	Pre-vs. post- (*P* value)	Peri- vs. post- (*P* value)
Age in years (mean, SD)	18	13	22	*P* = .0098	*P* = .0142	*P* = .0000
Sex (male %)	42.94	39.13	42.07	*P* = .279	*P* = .481	*P* = .335
Vaccination against COVID-19 (%)	0.00	1.23	12.80	*P* = .0000	*P* = .000	*P* = .0000

### 3.2 Respiratory symptoms and prevalence of pathogens

Of the pilgrims in the cohort, 431/837 (51.5 %) reported respiratory symptoms, including 319/837 (38.1 %) upon their return from the GMT. No statistically significant variations were observed for these symptoms according to the study periods. The most frequent symptoms on return were rhinitis, a cough, and sore throat. Febrile illnesses were reported by 119/837 (14.2 %) and 3/837 (0.4 %) of pilgrims were hospitalised. Overall, the prevalence of respiratory viruses upon return significantly decreased during the COVID period (9.5 %) compared to the pre-COVID and post-COVID periods (26.0 % and 21.5 %, respectively). The respiratory viruses most frequently found in pilgrims returning from the GMT were rhinovirus (10.3 %), followed by endemic coronaviruses (5.4 %), SARS-CoV-2 (1.7 %), and influenza A virus (1.6 %). The prevalence of rhinovirus significantly decreased during the COVID period (3.9 %) compared to the pre-COVID period (15.5 %) and post-COVID period (9.9 %). The prevalence of rhinovirus during the post-COVID period was still significantly lower than that of the pre-COVID period. A similar pattern was observed for endemic coronaviruses with prevalence decreasing from 9 % to 1 % during the COVID period and reverting to 4.3 % during the post-COVID period. SARS-CoV-2 cases were observed only in the post-COVID period. In contrast, influenza A virus prevalence was significantly higher during the COVID period (3.5 %) than during the pre- and post-COVID periods (0.3 % and 1.3 %). Overall, the prevalence of bacteria significantly decreased during and after the pandemic (74.5 % and 70.0 %, respectively, as compared to 85.2 % during the pre-COVID period). *K. pneumoniae* and *S. aureus* had a pattern similar to that of rhinovirus and endemic coronaviruses, with a significant decrease in their prevalence during the COVID period followed by a significant increase post-COVID albeit to levels significantly lower than during the pre-COVID period. In contrast, the prevalence of *Haemophilus* spp. increased significantly during the COVID period and reverted to pre-COVID levels during the post-COVID period. The prevalence of *M. catarrhalis* decreased over time during the whole study period, while that of *S. pneumoniae* remained stable over time (Table 3).

**Table 3:** Prevalence of respiratory pathogens in pilgrims from Ndiop and Dielmo upon returning from Touba (2017–2024).

	Pre-pandemic period (2017–2019)	Peri-pandemic period (2020–2021)	Post-pandemic period (2022–2024)	Pre- vs. peri- (*P* value)	Pre-vs. post- (*P* value)	Peri- vs. post- (*P* value)
A least one virus (%)	25.99	9.52	21.52	*P =* .000	*P =* .116	*P =* .000
Influenza A (%)	0.33	3.46	1.32	*P =* .006	*P =* .184	*P =* .088
Influenza B (%)	0.33	0.87	0.33	*P =* .398	*P =* .749	*P =* .400
SARS-CoV-2 (%)	0.00	0.00	4.97	–	*P =* .000	*P =* .000
Metapneumovirus (%)	0.00	0.43	0.66	*P =* .432	*P =* .248	*P =* .600
Respiratory syncytial virus (%)	0.00	0.43	0.99	*P =* .432	*P =* .123	*P =* .418
Human rhinovirus (%)	15.46	3.90	9.93	*P =* .000	*P =* .027	*P =* .005
Adenovirus (%)	2.30	0.00	0.99	*P =* .019	*P =* .173	*P =* .181
Human coronaviruses (%)	9.87	0.87	4.30	*P* = .000	*P* = .006	*P* = .014
A least one bacterium (%)	85.20	74.46	69.87	*P* = .001	*P* = .000	*P* = .142
*Haemophilus* spp*.* (%)	40.46	62.77	35.10	*P* = .000	*P* = .101	*P* = .000
*Klebsiella pneumoniae* (%)	43.75	1.30	15.89	*P* = .000	*P* = .000	*P* = .000
*Moraxella catarrhalis* (%)	11.84	15.58	19.87	*P* = .130	*P* = .005	*P* = .122
*Staphylococcus. aureus* (%)	45.07	17.32	24.83	*P* = .000	*P* = .000	*P* = .023
*Streptococcus pneumoniae* (%)	25.00	26.41	29.80	*P* = .393	*P* = .109	*P* = .222
Virus/bacteria (%)	20.72	9.09	19.87	*P* = .000	*P* = .436	*P* = .000

In Mbacké, 490 patients complained of respiratory symptoms, with all of them experiencing coughs, 338 (69 %) rhinitis, and 95 (19.4 %) sore throats. Two hundred and seven (42.2 %) had a febrile illness. Antibiotic treatment was given to 356/490 (72.7 %) and 40/490 (8.2 %) were hospitalised. The most commonly found respiratory viruses were influenza A virus (30.6 %), respiratory syncytial virus (12.9 %), rhinovirus (8.0 %), and influenza B virus (5.5 %). The prevalence of influenza A virus was significantly higher during and after the pandemic (34.57 % and 39.63 %) than before the pandemic (17.68 %). Overall, the prevalence of influenza B virus, HRV, and RSV decreased over time with significant variations when comparing the pre- and post-COVID periods. SARS-CoV-2 was only detected after the pandemic. Among bacteria, the prevalence of *S. pneumoniae* and *M. catarrhalis* significantly increased during the COVID period compared to before and after, while *S. aureus* prevalence significantly decreased during the COVID period. *K. pneumoniae* was rare before the pandemic, was not detected during the pandemic and significantly increased after the pandemic. In addition, the prevalence of *Haemophilus* spp. significantly decreased after the COVID period (Table 4).

**Table 4:** Prevalence of respiratory pathogens in patients consulting at the Mbacké health care centre.

	Pre-pandemic	Peri-pandemic	Post-pandemic	Pre- vs. peri- (*P* value)	Pre-vs. post- (*P* value)	Peri- vs. post- (*P* value)
A least one virus	61.59	64.20	57.32	*P =* .354	*P =* .250	*P =* .123
Influenza A	17.68	34.57	39.63	*P =* .000	*P =* .000	*P =* .203
Influenza B	9.15	4.94	2.44	*P =* .102	*P =* .008	*P =* .183
SARS-CoV-2	–	0.00	1.83	–	*P =* .124	*P =* .126
Metapneumovirus	1.83	4.32	1.83	*P =* .163	*P =* .658	*P =* .163
Respiratory syncitial virus	23.78	10.49	4.27	*P =* .001	*P =* .000	*P =* .025
Human rhinovirus	12.80	6.17	4.08	*P =* .031	*P =* .009	*P =* .394
Adenovirus	3.05	1.85	0.00	*P =* .368	*P =* .030	*P =* .122
Human coronaviruses	1.83	0.62	4.88	*P =* .316	*P =* .109	*P =* .019
Human parainfluenzae viruses	0.00	2.47	0.61	*P =* .060	*P =* .500	*P =* .182
A least one bacterium	94.51	87.65	61.59	*P =* .023	*P =* .000	*P =* .000
*Haemophilusinfluenzae*	75.61	69.75	27.44	*P =* .144	*P =* .000	*P =* .000
*Klebsiellapneumoniae*	1.83	0.00	10.37	*P =* .126	*P =* .001	*P =* .000
*MMoraxellacatarrhalis*	43.29	48.77	25.00	*P =* .189	*P =* .000	*P =* .000
*Staphylococcusaureus*	32.93	10.49	28.66	*P =* .000	*P =* .237	*P =* .000
*Streptococcuspneumoniae*	46.95	55.56	35.98	*P =* .074	*P =* .028	*P =* .000
Virus/bacteria	58.54	54.94	37.80	*P =* .293	*P =* .000	*P =* .001

### 3.3 Gastrointestinal symptoms and the prevalence of pathogens

Within the cohort, 102 (12.2 %) pilgrims reported gastrointestinal symptoms, 70 of whom reported symptoms upon their return from the GMT (8.4 % of all pilgrims). The most common symptoms upon return were abdominal pain 39/837 (4.7 %), diarrhoea 24/837 (2.9 %), and vomiting 16/837 (1.9 %). One pilgrim was hospitalised. The prevalence of gastrointestinal symptoms significantly decreased from 11.2 % before the pandemic to 6.5 % and 7.0 % during and after the pandemic (*P* = .04). The prevalence of intestinal bacteria, which was 55.6 % before the COVID pandemic, decreased significantly during the pandemic (34.6 %) and reverted after the pandemic to levels similar to that of the pre-pandemic period (52.0 %). This was mostly due to a significant decrease in *E. coli* pathotypes such as EAEC AND EIEC/Shigella during the pandemic, followed by a reversion to pre-pandemic levels. It was noted that EHEC and EPEC levels also decreased during the pandemic but remained at levels lower than those observed before the pandemic. *G. lamblia* was the only parasite detected and adenovirus was the only digestive virus identified. No statistically significant variations were observed for these parasitic and viral pathogens between the study periods (Table 5).

**Table 5:** Prevalence of gastrointestinal pathogens in pilgrims from Ndiop and Dielmo upon return from Touba.

	Pre-pandemic	Peri-pandemic	Post-pandemic	Pre- vs. peri- (*P* value)	Pre-vs. post- (*P* value)	Peri- vs. post- (*P* value)
At least one bacterium (%)	55.59	34.63	51.99	*P =* .000	*P =* .209	*P =* .000
EAEC (%)	38.16	30.74	42.72	*P =* .045	*P =* .145	*P =* .003
EHEC (%)	12.16	3.03	4.97	*P =* .000	*P =* .000	*P =* .186
EIEC/Shigella (%)	8.22	4.33	10.60	*P =* .050	*P =* .195	*P =* .005
EPEC (%)	30.59	2.16	5.63	*P =* .000	*P =* .000	*P =* .035
*Campylobacter jejuni* (%)	0.33	0.00	0.00	*P =* .568	*P =* .502	*P =* .418
*Tropheryma whipplei* (%)	4.28	1.73	0.66	*P =* .076	*P =* .003	*P =* .227
*Salmonella* spp (%)	2.63	0.00	0.00	*P =* .010	*P =* .004	–
At least one parasite (%)	2.96	5.19	5.63	*P =* .137	*P =* .077	*P =* .493
*Cryptosporidium* spp (%)	0.00	0.00	0.00	–	–	–
*Entamoeba histolytica* (%)	0.00	0.00	0.00	–	–	–
*Giardia lamblia* (%)	2.96	5.19	5.63	*P =* .137	*P =* .077	*P =* .493
At least one virus (%)	1.97	0.43	1.66	*P =* .119	*P =* .505	*P =* .183
Adenovirus (%)	1.97	0.43	1.66	*P =* .119	*P =* .505	*P =* .183
Hepatitis A virus (%)	0.00	0.00	0.00	–	–	–
HepatitisE virus (%)	0.00	0.00	0.00	–	–	–
Astrovirus (%)	0.00	0.00	0.00	–	–	–
Norovirus (%)	0.00	0.00	0.00	–	–	–
Rotavirus	0.00	0.00	0.00	–	–	–

EAEC: Enteroaggregative *Escherichia coli*, EHEC: Enterohemorrhagic *E. coli*, EIEC: Enteroinvasive *E. coli*, EPEC: Enteropathogenic *E. coli*.

## 4 Discussion

The COVID-19 pandemic has been associated with changes in respiratory virus infections worldwide, and these differences have varied between virus types. Reductions in respiratory virus infections, including influenza virus and respiratory syncytial virus, were most notable at the onset of the COVID-19 pandemic and continued in varying degrees through subsequent waves of SARS-CoV-2 infections [8]. According to the Respiratory Virus Global Epidemiology Network, distinct sequential timing in the first post-pandemic resurgence of respiratory viruses was observed. Rhinovirus resurged the earliest, followed by seasonal coronavirus, parainfluenza virus, respiratory syncytial virus, adenovirus, metapneumovirus, and influenza A virus, with influenza B virus exhibiting the latest resurgence [9]. These seasonal disruptions of viral pathogens were shown to correlate with the degree of non-pharmaceutical interventions against COVID-19 [10]. Before the COVID-19 pandemic, influenza activity in Senegal followed a normal pattern, with low circulation throughout the year and a peak during the rainy seasons between August and October and another between January and March [11]. In our study, we observed a significant decrease in the prevalence of certain viruses including, notably, rhinovirus and endemic coronaviruses, but a marked increase in influenza A cases during the COVID-19 pandemic. This was not the case in certain countries in the southern hemisphere, such as Australia and South Africa, where low influenza virus circulation was noted during the same period [12]. The same phenomenon was observed in the northern hemisphere with the absence of the seasonal flu peak in the winter of 2020–2021 [13,14]. A low circulation of influenza viruses was noted between 2020 and 2021 in a study conducted in Gabon [15]. In Senegal, a national study identified a change in the epidemiological profile of influenza in 2021 and 2022 with an absence of peak between January and March, while an unusual peak was observed between May and July 2022.

According to various studies, the impact of the COVID-19 pandemic on bacterial acute respiratory disease varied. Marked reductions in invasive *S. pneumoniae* and *S. pyogenes* and *H. influenzae* were observed, followed by a resurgence that correlated with increases in respiratory viral infections [16,17]. Our observations at the GMT were different, with the prevalence of *S. pneumoniae* and *M. catarrhalis* significantly increasing during the COVID period, while that of *S. aureus* significantly decreased.

Finally, the incidence of other pathogens, including gastrointestinal pathogens, was also affected by the pandemic. Overall, a larger reduction was observed for viral gastrointestinal infections compared with bacterial gastrointestinal infections during the COVID-19 pandemic, particularly for norovirus. For bacterial gastrointestinal infections, *Campylobacter* and non-typhoidal *Salmonella* were the most frequently detected pathogens in the majority of the studies, with the largest reduction being observed for *Shigella* and Shiga toxin–producing *Escherichia coli* infections [18]. In our experience at the GMT, the prevalence of several *E. coli* pathotypes decreased during the pandemic.

Comparing our results to other studies is hazardous since, firstly, we only investigated a short period of time each year and, secondly, the specific context of the mass gathering with participants from all Senegal and other countries in Africa and beyond is likely to have influenced the circulation of respiratory and gastrointestinal pathogens. It should also be noted that most studies addressing the global impact of the COVID-19 pandemic on other infections were conducted in countries outside Africa.

In conclusion, significant variations in the prevalence of respiratory and gastrointestinal pathogens were observed in pilgrims participating in the GMT during the COVID-19 pandemic in Senegal. Given that the major behavioural changes documented in our study included an increased use of face masks and soap for hand washing, this could possibly have impacted the circulation of certain pathogens in the context of the GMT. However, demographic changes in the participants were also observed during the pandemic which could also have had an impact on the local epidemiology of respiratory and gastrointestinal infections at the GMT.

## CRediT authorship contribution statement

**Coumba Diouf:** Writing – review & editing, Writing – original draft, Methodology, Investigation. **Ihssane Ouaddane:** Validation, Methodology. **Ndiaw Goumballa:** Validation, Methodology. **Déguéne Fam:** Methodology. **Hubert Bassene:** Methodology. **Cheikh Sokhna:** Supervision, Project administration, Funding acquisition, Conceptualization. **Philippe Gautret:** Writing – review & editing, Validation, Supervision, Project administration, Funding acquisition, Conceptualization.

## Ethics

The protocol was approved by the National Ethics Committee for Health Research in Senegal (SEN17/62) and performed in accordance with the good clinical practices recommended by the Declaration of Helsinki and its amendments.

## Consent for publication

All authors contributed to and approved the current version of the manuscript.

## Funding

This study was supported by the Institut Hospitalo-Universitaire (IHU) Méditerranée Infection, and the French National Research Agency under the ‘Investissements d’Avenir’ program, reference ANR-10-IAHU-03.

## Declaration of competing interest

The authors declare that they have no known competing financial interests or personal relationships that could have appeared to influence the work reported in this paper.

## References

[1] Sokhna C., Mboup B.M., Sow P.G., Camara G., Dieng M., Sylla M., Gueye L., Sow D., Diallo A., Parola P., Raoult D., Gautret P. (2017 Sep). Communicable and non-communicable disease risks at the grand magal of touba: the largest mass gathering in Senegal. Trav Med Infect Dis.

[2] Sokhna C., Goumballa N., Hoang V.T., Mboup B.M., Dieng M., Sylla A.B., Diallo A., Raoult D., Parola P., Gautret P. (2020 Feb). Senegal's grand magal of touba: syndromic surveillance during the 2016 mass gathering. Am J Trop Med Hyg.

[3] Goumballa N., Hoang V.T., Diouf F.S., Mbaye B., Parola P., Sokhna C., Gautret P. (2022 Sep-Oct). Risk factors for symptoms of infection and the acquisition of pathogens among pilgrims at the grand magal of touba, 2017-2021. Trav Med Infect Dis.

[4] Goumballa N., Sambou M., Samba D.F., Bassene H., Bedotto M., Aidara A., Dieng M., Hoang V.T., Parola P., Sokhna C., Gautret P. (2023 Mar-Apr). PCR investigation of infections in patients consulting at a healthcare centre over a four-year period during the Grand Magal of Touba. Trav Med Infect Dis.

[5] Huang C., Wang Y., Li X., Ren L., Zhao J., Hu Y., Zhang L., Fan G., Xu J., Gu X., Cheng Z., Yu T., Xia J., Wei Y., Wu W., Xie X., Yin W., Li H., Liu M., Xiao Y., Gao H., Guo L., Xie J., Wang G., Jiang R., Gao Z., Jin Q., Wang J., Cao B. (2020 Feb 15). Clinical features of patients infected with 2019 novel coronavirus in Wuhan, China. Lancet.

[6] Dia N., Lakh N.A., Diagne M.M., Mbaye K.D., Taieb F., Fall N.M., Barry M.A., Ka D., Fall A., Diallo V.M.P.C., Faye O., Jallow M.M., Dieng I., Ndiaye M., Diop M., Bousso A., Loucoubar C., Ndiaye M.K.N., Peyreffite C., Fortes L., Sall A.A., Faye O., Seydi M. (2020 Nov). COVID-19 Outbreak, Senegal, 2020. Emerg Infect Dis.

[7] Ndiaye M., Sow K.D., Ly A.B., Diop B., Ba M., Faye A. (2022 Dec 23). Epidemiology and response strategies against COVID-19: the Senegalese experience from 2020 to 2021. Pan Afr Med J.

[8] Chow E.J., Uyeki T.M., Chu H.Y. (2023 Mar). The effects of the COVID-19 pandemic on community respiratory virus activity. Nat Rev Microbiol.

[9] Zhao C., Zhang T., Guo L., Sun S., Miao Y., Yung C.F., Tomlinson J., Stolyarov K., Shchomak Z., Poovorawan Y., Nokes D.J., Muñoz-Almagro C., Mandelboim M., Keck J.W., Langley J.M., Heikkinen T., Deng J., Colson P., Chakhunashvili G., Caballero M.T., Bont L., Feikin D.R., Nair H., Wang X., Li Y. (2025 Feb 13). Respiratory Virus Global Epidemiology Network. Characterising the asynchronous resurgence of common respiratory viruses following the COVID-19 pandemic. Nat Commun.

[10] Eggeling R., König F., Koeppel L., Böhler L.I., Böhm M., Schmeißer N., Pfeifer N., Kaiser R. (2025 Aug 5). Clinical Virology Network. Long-term impact of the SARS-CoV-2 pandemic on respiratory viruses in Germany. BMC Public Health.

[11] Lampros A., Talla C., Diarra M., Tall B., Sagne S., Diallo M.K., Diop B., Oumar I., Dia N., Sall A.A., Barry M.A., Loucoubar C. (2023 Sep). Shifting patterns of influenza circulation during the COVID-19 pandemic, Senegal. Emerg Infect Dis.

[12] Olsen S.J., Azziz-Baumgartner E., Budd A.P., Brammer L., Sullivan S., Pineda R.F., Cohen C., Fry A.M. (2020 Sep 18). Decreased Influenza Activity during the COVID-19 Pandemic - united States, Australia, Chile, and South Africa, 2020. MMWR Morb Mortal Wkly Rep.

[13] Solomon D.A., Sherman A.C., Kanjilal S. (2020 Oct 6). Influenza in the COVID-19 era. JAMA.

[14] Olsen S.J., Winn A.K., Budd A.P., Prill M.M., Steel J., Midgley C.M., Kniss K., Burns E., Rowe T., Foust A., Jasso G., Merced-Morales A., Davis C.T., Jang Y., Jones J., Daly P., Gubareva L., Barnes J., Kondor R., Sessions W., Smith C., Wentworth D.E., Garg S., Havers F.P., Fry A.M., Hall A.J., Brammer L., Silk B.J. (2021 Jul 23). Changes in influenza and other respiratory virus activity during the COVID-19 pandemic - united States, 2020-2021. MMWR Morb Mortal Wkly Rep.

[15] Moulengui C.K., Lekana-Douki J.B., Lekana-Douki S.E. (2025 Jul 2). The impact of the COVID-19 pandemic on other respiratory viruses in the Haut-Ogooue Province of Gabon. IJID Reg.

[16] Burrell R., Saravanos G., Britton P.N. (2025 Mar). Unintended impacts of COVID-19 on the epidemiology and burden of paediatric respiratory infections. Paediatr Respir Rev.

[17] Kajihara T., Yahara K., Kamigaki T., Hirabayashi A., Hosaka Y., Kitamura N., Shimbashi R., Suzuki M., Sugai M., Shibayama K. (2024 Aug). Effects of coronavirus disease 2019 on the spread of respiratory-transmitted human-to-human bacteria. J Infect.

[18] Lazarakou A., Mughini-Gras L., Pijnacker R. (2024 Nov 26). Global impact of COVID-19 pandemic on gastrointestinal infections: a scoping review. Foodborne Pathog Dis.

